# Preterm Birth Has Sex-Specific Effects on Autonomic Modulation of Heart Rate Variability in Adult Sheep

**DOI:** 10.1371/journal.pone.0085468

**Published:** 2013-12-26

**Authors:** Mary Berry, Anne Jaquiery, Mark Oliver, Jane Harding, Frank Bloomfield

**Affiliations:** 1 Liggins Institute, University of Auckland, Auckland, New Zealand; 2 Gravida: National Centre for Growth and Development, University of Auckland, Auckland, New Zealand; 3 Department of Paediatrics: Child and Youth Health, University of Auckland, Auckland, New Zealand; Université de Montréal, Canada

## Abstract

**Introduction:**

Globally, 11% of infants are born preterm. In adulthood, individuals born preterm are at increased risk of cardiovascular morbidity and mortality, but the mechanistic basis of this remains unknown. Clinically overt cardiovascular disease may be preceded by altered cardiac autonomic activity characterised by increased sympathetic activity and/or reduced parasympathetic activity. Thus, altered cardiac autonomic activity in survivors of preterm birth may underlie later cardiovascular risk.

**Objective:**

To investigate the impact of gestational age on cardiac autonomic activity in juvenile and adult sheep.

**Methods and Results:**

Singleton-bearing ewes were randomised antenatally to spontaneous term birth (TC; n=73) or corticosteroid induced preterm birth (PT; n=60). Cardiac autonomic modulation was assessed using heart rate variability analysis in juvenile and adult offspring. Preterm birth in adult males was associated with altered sympatho-vagal modulation (LFnu: PT 64±4 vs. TC 49±4, p<0.05; LogLF/HF: PT 1.8±0.1 vs. TC 1.5±0.1, p<0.05) and reduced parasympathetic modulation (LogRMSSD: PT 2.9±0.2 vs. TC 3.4±0.1, p<0.05; LogNN50: PT 0.3±0.4 vs. TC 1.6±0.4, p<0.05). Within the range of term birth, each one-day increment in gestational age was associated with a decrement in LFnu in juvenile females and with a decrement in LFnu and LF/HF ratio, but an increment in RMSSD and NN50 in adult females.

**Conclusions:**

Cardiac autonomic function in adult sheep is affected in a sex-specific manner by gestational age at birth, even within the term range. Altered cardiac autonomic function may contribute to increased later cardiovascular morbidity in those born preterm.

## Introduction

As preterm birth rates and survival following preterm birth increase, it is becoming increasingly apparent that preterm birth is accompanied by later cardiovascular morbidity: increased blood pressure [1,2], altered cardiovascular structure and function [3,4] and increased mortality [5]. Determining mechanisms is hampered by the latency between preterm birth and later morbidity and the complex socioeconomic factors that frequently co-exist in this population [6]. However, even small reductions in gestation length within the term gestational range are associated with later medical and cognitive disadvantage [7,8] and with sex-specific changes in blood pressure [9], suggesting that duration of gestation is itself a key factor. 

The fetal autonomic nervous system increases in complexity as gestation advances, with reducing sympathetic and increasing parasympathetic activity [10]. In the ovine fetus, maturation of parasympathetic activity occurs during late gestation and appears to be related to fetal growth [11]. Preterm birth disrupts the normal late-gestation trajectory of growth and organ maturation, and is associated with altered autonomic balance in human infants [12,13] and in newborn experimental animals [14]. 

We hypothesized that gestation length at birth would alter autonomic balance in juvenile and adult sheep and that this might provide a mechanistic basis for increased cardiovascular morbidity in those born preterm. 

## Methods

Ethics statement: Ethical approval for this study was obtained prospectively from the University of Auckland Animal Ethics Committee (AEC 628). 

Time-mated, singleton-bearing Romney ewes (n=133) were generated [15]. At day 131 of a 147-day pregnancy ewes were randomised to spontaneous labour at term (term-control; TC) or preterm induction of labour (PT). 

PT ewes received 0.25mg/kg dexamethasone sodium phosphate (Dexa 0.2, Southern Veterinary Supplies, Hamilton, NZ) by intramuscular injection on days 135 and 136 with birth of the lamb on d137. 

TC ewes received no interventions during pregnancy. 

At 5 months’ (juvenile) and 16 months’ (adult) corrected postnatal age, electrocardiograms (ECGs) were obtained using a PowerLab^®^ system (ADInstruments, Dunedin, NZ) from standing, conscious sheep in a quiet, temperature and light-controlled (18°C, lights on 0700, lights off 1900) environment to which they had been extensively habituated. To control for any effect of diurnal variation in autonomic tone [16], all ECGs were obtained in the early afternoon. To control for variation in oestrous cycle, adult females had intravaginal progesterone-containing controlled release device (ovine CIDR, Southern Veterinarian Supplies, Hamilton, NZ) placed at least 3 days prior to testing. 

Animals were studied while housed in metabolic crates to which they were also well habituated, with standard sheep pellets and water available *ad libitum*. Subcutaneous ECG needles (MLA1204, ADInstruments, Dunedin, NZ) were placed on the sternum, over the left scapula and the left flank and secured in place with adhesive [17]. After a one hour non-intervention period following needle placement a one hour ECG was recorded (sample rate 1k/s). A five minute section of the ECG was selected based on signal quality and absence of movement artefact and analysed using LabChart 7 software (ADInstruments, Dunedin, NZ) for heart rate (HR) and measures of heart rate variability (HRV): RMSSD (the square root of the mean of the sum of the squares of the successive differences between adjacent normal to normal (NN) beats); NN50 (the number of pairs of successive NN intervals that differ by more than 50 ms) and the frequency domain parameters of low frequency (LF; 0.04-0.15 Hz), low frequency expressed relative to the total power of the ECG (LFnu), high frequency (HF 0.15-0.4 Hz), and the LF/HF ratio [18]. These frequency bandwidths correspond to those used in the assessment of HRV in adult large animals [19], reflecting the lower respiratory and heart rate of adults compared to the fetus when different bandwidths are more appropriate [11]. ECGs were obtained from the whole cohort and the quality of the trace manually verified. All those ECGs free of movement artefact or other signal degradation were analysed and reported here. 

As cardiovascular disease risk is known to be different between the sexes, males and females were analysed separately using JMP v. 8 (SAS Institute Inc., Cary, NC, USA). Non-parametric data were log transformed prior to analysis. Data were analysed by factorial ANOVA with Tukey post hoc correction. Associations between continuous variables were examined by regression analysis within each birth group, in same sex animals. 

Multivariate regression models were adjusted for the effect of: birthweight z-score (birthweight - mean birthweight for gestation and sex / SD of mean birthweight for gestation and sex), age and weight (juveniles) or weight-for-length (weight (kg) / crown-rump length (m)) in adults as a better measure of soft tissue mass relative to skeletal growth. Measures of length were not available for juvenile sheep. 

## Results

At birth, but not in juvenile or adult life, same-sex PT lambs were smaller and lighter than TC lambs (Table 1). 

**Table 1:** Anthropometric characteristics of sheep.

	Male		Female	
	Term	Preterm	Term	Preterm
**Birth**				
N	39	30	34	30
Gestational age (days)	147.8 ± 0.3	137.3 ± 0.3**	147.2 ± 0.3	137.3 ± 0.3**
Weight (kg)	6.1 ± 0.1	5.0 ± 0.1**	5.6 ± 0.1	4.6 ± 0.1**
Birthweight z-score	0.3 ± 0.2	0.1 ± 0.2	0.3 ± 0.2	0.1 ± 0.3
**Juvenile**				
N	19	14	14	15
Age (weeks)	17.3 ± 0.5	17.8 ± 0.6	18.6 ± 0.5	18.3 ± 0.6
Weight (kg)	35.1 ± 1.0	35.5 ± 1.1	33.6 ± 1.0	33.3 ± 1.1
**Adult**				
N	23	22	22	17
Age (months)	16.1 ± 0.2	15.9 ± 0.3	15.9 ± 0.3	15.3 ± 0.3
Weight (kg)	67.5 ± 1.9	70.6 ± 2.0	60.1 ± 1.5	56.3 ± 1.6
Weight-for-length (Kg/m)	59.8 ± 1.6	60.8 ± 1.7	54.5 ± 1.2	51.3 ± 1.3

Data are mean ± SEM. ** p<0.01 preterm compared to term control.

In males, PT sheep had altered indices of sympatho-vagal modulation as juveniles and adults, with reduced indices of parasympathetic modulation in adulthood, but no difference in heart rate at either age (Table 2). In females, PT sheep had no differences in measures of autonomic balance at either age but had a higher heart rate as juveniles and a lower heart rate as adults (Table 2). 

**Table 2:** Effect of preterm birth on heart rate variability parameters.

	**Male**			**Female**		
**Juvenile**	Term	Preterm	p-value	Term	Preterm	p-value
N	19	14		14	15	
HR (bpm)	101 ± 7	118 ± 7	0.09	99 ± 6	121 ± 5	0.02
LFnu	39 ± 4	59 ± 5	<0.01	41 ± 8	40 ± 6	0.90
Log LF/HF	0.3 ± 0.2	1.0 ± 0.2	0.02	0.5 ± 0.3	0.3 ± 0.2	0.63
Log RMSSD	3.4 ± 0.2	2.9 ± 0.2	0.08	3.7 ± 0.3	3.3 ± 0.3	0.42
Log NN50	1.4 ± 0.5	0.1 ± 0.5	0.09	2.0 ± 0.7	1.5 ± 0.6	0.59
**Adult**						
N	23	22		22	17	
HR (bpm)	101 ± 4	112 ± 5	0.09	108 ± 3	100 ± 3	0.04
LFnu	49 ± 4	64 ± 4	0.02	55 ± 5	49 ± 6	0.48
Log LF/HF	1.5 ± 0.1	1.8 ± 0.1	0.04	1.6 ± 0.1	1.6 ± 0.1	0.79
Log RMSSD	3.4 ± 0.1	2.9 ± 0.2	0.01	3.2 ± 0.2	3.5 ± 0.2	0.35
Log NN50	1.6 ± 0.4	0.3 ± 0.4	0.03	1.3 ± 0.4	1.7 ± 0.5	0.49

Data are mean ± SEM.

**Figure 1:**
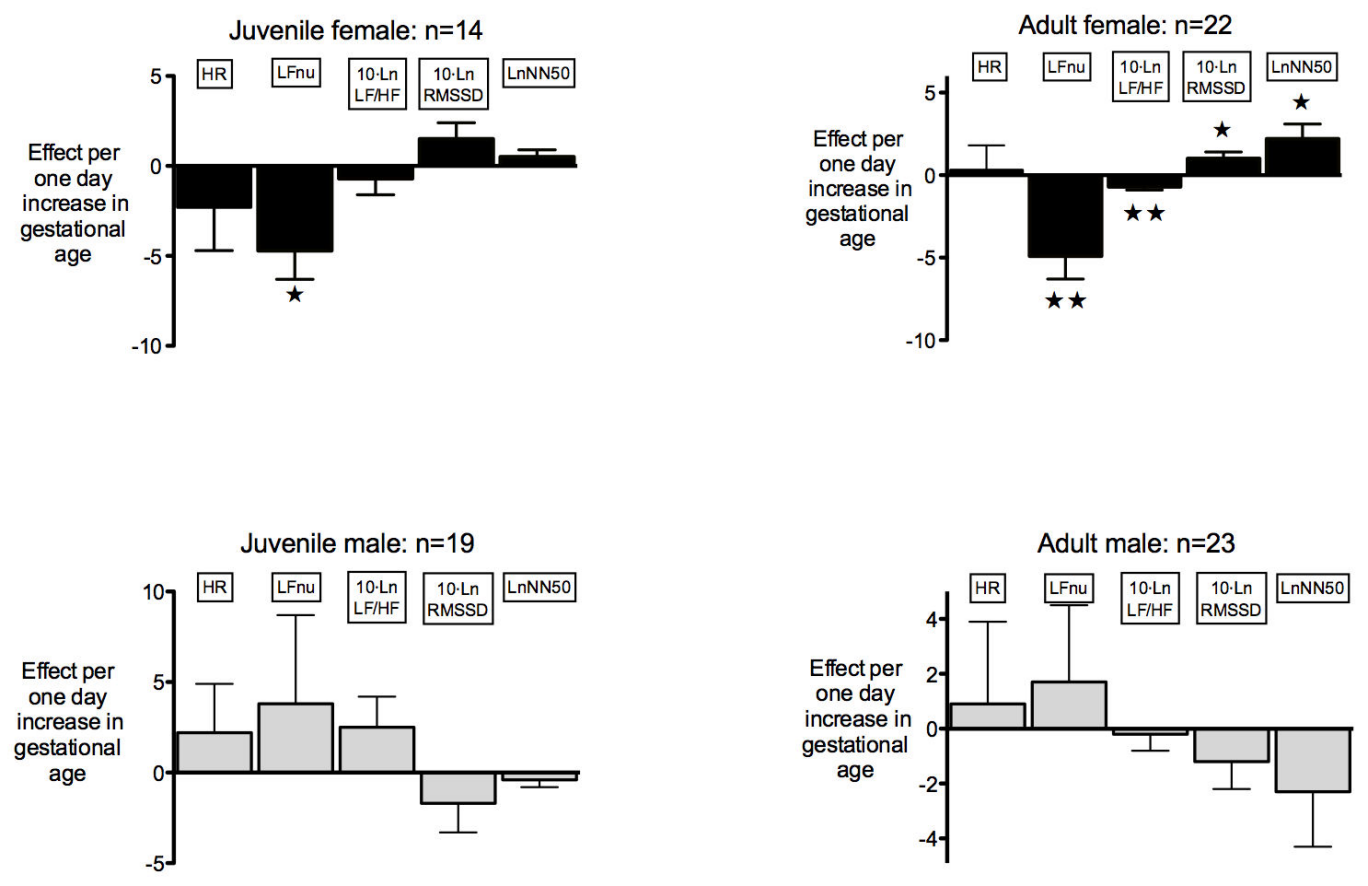
The effect of gestational age across the term range on heart rate variability parameters. Data are mean ± SEM. *P<0.05; **P<0.01.

TC sheep had a gestational age range of 142-156 days (male 144-153 days, female 142-156 days). In females, each one-day increment in gestational age was associated in juveniles with a decrement in LFnu and in adulthood with a decrement in LFnu and LF/HF ratio, but an increment in RMSSD and NN50 (Figure 1). 

## Discussion

Our data demonstrate that both preterm birth and gestation length within the term range result in sex-specific effects on autonomic balance in juvenile and adult sheep, with reduced gestation length associated with altered sympatho-vagal balance. 

Although HRV does not represent direct measurements of autonomic nerve activity, it is a sensitive technique for estimation of overall autonomic balance and is predictive of cardiovascular morbidity. Tonic elevation of sympathetic activity and / or parasympathetic withdrawal may precede development of clinically overt hypertension [20,21] and cardiac hypertrophy [22], alter angiogenesis and vascular remodelling [23,24], contribute to the evolution of diabetes [25,26] and underlie sudden cardiac death [27]. The altered sympatho-vagal balance that we report here may therefore underlie the association between preterm birth and these later adverse cardiovascular outcomes [2,4,5,28,29]. 

Gestational age and preterm corticosteroid exposure both may be implicated in early postnatal autonomic function and separation of the two factors is challenging. Steroid-naïve preterm birth in experimental animals is associated with significant mortality [30] and, in humans, corticosteroid treatment to women at risk of preterm birth is gold-standard care [31]. Our approach, combining corticosteroid exposure with preterm birth, is therefore a relevant experimental paradigm. 

Preterm birth in lambs is not accompanied by the increase in HR, BP and renal sympathetic nerve activity observed in term-born lambs unless the ewe receives antenatal corticosteroid treatment [32]. However, our observation of a sex-specific effect of gestational age at term on later autonomic balance in steroid-naïve animals suggests that exogenous corticosteroid exposure *per se* is unlikely to be the sole mediator of the differences in HRV between preterm- and term-born sheep. 

The emergence of sexually dimorphic HRV characteristics is congruent with the sex-specific differences in manifestation of cardiovascular disease [33]. Sexually dimorphic modulation of autonomic tone has been described in humans and animals [34,35] and, in rats, has been linked to oestrus-related fluctuation in sex-hormones [36]. Our preterm and term-born females were therefore studied with progesterone-secreting CIDRs *in situ* to control for the effect of variation in oestrous cycling between animals. The impact of gestational age at birth on age-related changes in sex hormones was not assessed in this study, but may be one mechanism through which gestational age modifies later sympatho-vagal balance. Further studies are needed to define the interaction between normal cyclical changes in sex hormones in animals born preterm on modulation of autonomic tone. It is also possible that in our female sheep the differences in baseline heart rate masked differences in HRV [37]. However, the absolute difference in average heart rate was small between preterm and term-born animals, and the range in heart rate was comparable between all groups. It is therefore unlikely that such small differences in average heart rate were themselves sufficient to mask any effect of preterm birth on HRV in females. With respect to the effect of gestational age across the term continuum on HRV, it is possible that the apparent absence of an effect in males is due to their narrower gestational age range compared to the females, which might have reduced the power of the analysis to detect an effect.

Our data describe the effect of gestational age and preterm birth in singleton animals only. In humans, although preterm birth is common in twins and higher order multiples, singletons account for approximately 75% of the preterm population [38]. Differences in autonomic maturation between twins and singletons have been described in near-term fetal sheep and appear to be due to the different growth rates found in singleton compared to twin gestation [11]. Additionally, unlike as is the case in humans, preterm delivery was imposed on a healthy ewe with a normal pregnancy and normally grown fetus that would otherwise have delivered at term. By limiting our study to singleton offspring from pregnancies free from maternal or fetal co-morbidity, it is possible to isolate the late effects of gestation length from other potentially confounding factors.

Finally, the large number of animals studied is a strength of this study but also limited us to the use of a tethered ECG system with relatively short ECG recordings compared to those that can be acquired through a telemetric system. Future studies will include ECG recordings throughout a 24 hour period and also blood pressure recordings to better define the low frequency modulation of HRV and better characterise the contribution of the sympathetic nervous system to overall autonomic balance. Despite these limitations, we believe that these data represent a novel insight into a late autonomic dysfunction arising from preterm birth. 

## Conclusion

Preterm birth in sheep results in altered sympatho-vagal modulation of autonomic activity in adulthood in a sex-specific manner. This may contribute to the emergence of adult cardiovascular morbidity in humans born preterm.
